# Predictive Value of Serial Rapid Shallow Breathing Index Measurements for Extubation Success in Intensive Care Unit Patients

**DOI:** 10.3390/medicina60081329

**Published:** 2024-08-16

**Authors:** Semin Turhan, Duygu Tutan, Yeliz Şahiner, Alperen Kısa, Sibel Önen Özdemir, Mehmet Berksun Tutan, Selçuk Kayır, Güvenç Doğan

**Affiliations:** 1 Department of Anesthesiology and Reanimation, Erol Olçok Training and Research Hospital, 19040 Çorum, Turkey; sibelonen89@gmail.com; 2 Department of Internal Medicine, Hitit University Faculty of Medicine, 19040 Çorum, Turkey; duygus_781@hotmail.com; 3 Department of Anesthesiology and Reanimation, Medical Point Hospital, 27090 Antep, Turkey; yelizsahiner@gmail.com; 4 Department of Anesthesiology and Reanimation, Hitit University Faculty of Medicine, 19040 Çorum, Turkey; alperenkisa@hotmail.com (A.K.); drskayir@gmail.com (S.K.); guvencdogan@gmail.com (G.D.); 5 Department of General Surgery, Alaca State Hospital, 19600 Çorum, Turkey; mbtutan@gmail.com

**Keywords:** serial rapid shallow breathing index, intensive care unit, mechanical ventilation, extubation success, predictive value

## Abstract

*Background and Objectives*: Extubation success in ICU patients is crucial for reducing ventilator-associated complications, morbidity, and mortality. The Rapid Shallow Breathing Index (RSBI) is a widely used predictor for weaning from mechanical ventilation. This study aims to determine the predictive value of serial RSBI measurements on extubation success in ICU patients on mechanical ventilation. *Materials and Methods*: This prospective observational study was conducted on 86 ICU patients at Hitit University between February 2024 and July 2024. Patients were divided into successful and unsuccessful extubation groups. RSBI values were compared between these groups. *Results*: This study included 86 patients (32 females, 54 males) with a mean age of 54.51 ± 12.1 years. Extubation was successful in 53 patients and unsuccessful in 33. There was no significant difference in age and intubation duration between the groups (*p* = 0.246, *p* = 0.210). Significant differences were found in RSBI-1a and RSBI-2 values (*p* = 0.013, *p* = 0.011). The median RSBI-2a was 80 in the successful group and 92 in the unsuccessful group (*p* = 0.001). The ΔRSBI was higher in the unsuccessful group (*p* = 0.022). ROC analysis identified optimal cut-off values: RSBI-2a ≤ 72 (AUC 0.715) and ΔRSBI ≤ −3 (AUC 0.648). RSBI-2a ≤ 72 increased the likelihood of successful extubation by 10.8 times, while ΔRSBI ≤ −3 increased it by 3.4 times. Using both criteria together increased the likelihood by 28.48 times. *Conclusions*: Serial RSBI measurement can be an effective tool for predicting extubation success in patients on IMV. These findings suggest that serially measured RSBI may serve as a potential indicator for extubation readiness.

## 1. Introduction

Invasive mechanical ventilation (IMV) is a critical procedure for managing respiratory dynamics after securing the airway through procedures like endotracheal intubation or emergency tracheostomy. Reducing the duration of IMV can significantly lower ventilator-associated complications, morbidity, mortality, and healthcare costs [1]. Effective airway management and respiratory support are crucial in both Intensive Care Units (ICU) and Respiratory Care Units (RCU) [2].

Weaning patients from mechanical ventilation to spontaneous breathing is a crucial process in ICU and RCU settings. Delayed weaning can increase the risk of ventilator-associated pneumonia and other adverse effects, prolong ICU stays, or even lead to patient mortality. Therefore, daily assessment of a patient’s readiness to discontinue IMV is essential, involving parameters such as FiO_2_ < 0.5, PEEP ≤ 5, PaO_2_/FiO_2_ > 200, respiratory rate < 30/min, absence or reduction of IMV need, consciousness or cough reflex, hemodynamic stability, and stable metabolic functions [3]. Additionally, the ability to protect the airway, the amount of airway secretions, cough strength, and level of consciousness must be evaluated [4].

Despite the global use of these criteria, approximately 2% of extubations fail, necessitating the re-initiation of IMV [5]. Successful extubation is defined as a patient passing a spontaneous breathing trial (SBT), being extubated following the SBT, and not requiring reintubation within the subsequent 48 h.

Several scoring systems have been proposed to identify patients who can be successfully weaned and extubated from IMV [6]. However, there are noticeable differences among studies.

The Rapid Shallow Breathing Index (RSBI), first introduced by Yang and Tobin, is one of the most commonly used indices [7]. RSBI is calculated using the formula: respiratory rate/tidal volume. Various studies have reported different specificities and sensitivities for RSBI [8,9].

This study aims to define the specificity, sensitivity, negative predictive value, and positive predictive value of RSBI during SBT and compare these values with those reported in previous studies. Additionally, this research investigates the potential of serial RSBI measurements as a predictive tool for extubation success in ICU patients.

## 2. Materials and Methods

### 2.1. Patient Enrollment

This prospective observational study was conducted in the ICU of Hitit University Faculty of Medicine joint hospital between February 2024 and July 2024. The ICU has a capacity of 80 beds and typically treats patients with conditions such as sepsis, pneumonia, heart failure, COPD exacerbation, trauma, and other life-threatening pathologies. This study was approved by the institutional ethics committee (protocol number: 2023-117, date: 1 November 2023) and was conducted in accordance with the Helsinki Declaration. Written informed consent was obtained from the relatives of all patients prior to participation. Patients under 18 years of age, those with neuromuscular diseases, those who had used neuromuscular blocking agents in the last 48 h, those with diaphragm dysfunction, thoracostomy, pneumothorax, pneumomediastinum, obesity (body mass index (BMI) ≥ 30 kg/m^2^), pregnancy, and known heart failure were excluded from the study. Inclusion criteria were the absence of IMV need, inspiratory oxygen fraction (FiO_2_) ≤ 0.5, oxygen saturation (SaO_2_) > 90%, PaO_2_/FiO_2_ > 200, positive end-expiratory pressure (PEEP) < 5 cmH_2_O, respiratory rate < 30 breaths/min, normal acid–base balance, no inotropic support or no increase in inotropic support in the last 24 h, heart rate < 120 bpm, and systolic blood pressure between 90–160 mmHg [10].

### 2.2. Study Design

Patients meeting the inclusion criteria underwent spontaneous breathing trials (SBTs). In the first stage, patients were placed on pressure support (PS) ventilation mode with continuous positive airway pressure (CPAP) mode, PS of 5 cmH2O, and PEEP of 5 cmH_2_O. The ventilator used was Dräger Evita^®^ Infinity^®^ V500 Ventilation Unit. Patients with RSBI > 105 were returned to mechanical ventilation, and RSBI was recalculated the next day. Patients with RSBI < 105 were included in the study. In the absence of adverse changes such as sweating, respiratory rate > 37 breaths/min, hemodynamic instability (heart rate > 140, systolic blood pressure ≥ 180 mmHg and <90 mmHg), PaCO_2_ > 50, increase in PaCO_2_ by more than 7 mmHg, pH < 7.33, and increased respiratory workload, the second stage was initiated without pressure support. Extubation failure was defined as the need for reintubation or non-invasive mechanical ventilation (NIMV) within 48 h post-SBT.

### 2.3. Statistical Analysis

Data analysis was performed using SPSS version 23 software. Frequency and percentage were used for categorical variables, while mean and standard deviation (SD) were used for continuous variables. Quantitative data were presented as mean, median, SD, and range (minimum–maximum). The difference between RSBI-1 measured at the beginning of the procedure and RSBI-2a measured before extubation was calculated using the formula ΔRSBI = [RSBI-2a] − [RSBI-1]. Student’s *t*-test was used for parametric data, and the Mann–Whitney U test was used for non-parametric data. A ROC (Receiver Operating Characteristic) curve analysis was performed to evaluate diagnostic accuracy. The area under the ROC curve (AUC) was calculated to determine the optimal cut-off values for the variables. Sensitivity, specificity, positive predictive value, and negative predictive value of the cut-off values were calculated to assess diagnostic power. Approximate odds ratios were calculated using the Haldane–Anscombe method for calculations where the probability ratio approached infinity. Statistical significance was considered at *p* < 0.05. Based on the study by Song J. et al. [11], a power analysis was conducted, and the sample size was determined to be 49 patients with 0.80 power and 0.95 error.

## 3. Results

A total of 86 patients were included in this study. Table 1 summarizes the baseline characteristics of the patients. Of the included patients, 32 were female (37.2%) and 54 were male (62.8%). The mean age of the patients was 54.51 ± 12.1 years. There were 53 patients with successful extubation and 33 with unsuccessful extubation. There was no statistically significant difference between the groups in terms of age and duration of intubation (*p* = 0.246, *p* = 0.210, respectively).

Comorbidities of the patients are shown in Table 2, with pneumonia being the most common (34.9%) and sepsis the least common comorbidity (10.5%). The duration of IMV ranged from 2 to 48 days, with a mean of 10.67 ± 9.8 days. After the SBT, 53 patients (61.6%) had successful extubation, while 33 patients (38.4%) had unsuccessful extubation.

RSBI values in patients with successful and unsuccessful extubation are shown in Table 3. There was no significant difference in RSBI-1 values between the groups (*p* = 0.320). However, there were significant differences in RSBI-1a and RSBI-2 values (*p* = 0.013, *p* = 0.011, respectively). The median RSBI-2a was 80 (54–97) in the successful extubation group, while it was 92 (65–120) in the unsuccessful extubation group (*p* = 0.001). The mean ΔRSBI value was significantly higher in the unsuccessful extubation group (14.06 ± 15.15) compared to the successful extubation group (4.83 ± 17.87) (*p* = 0.022).

**Figure 1:**
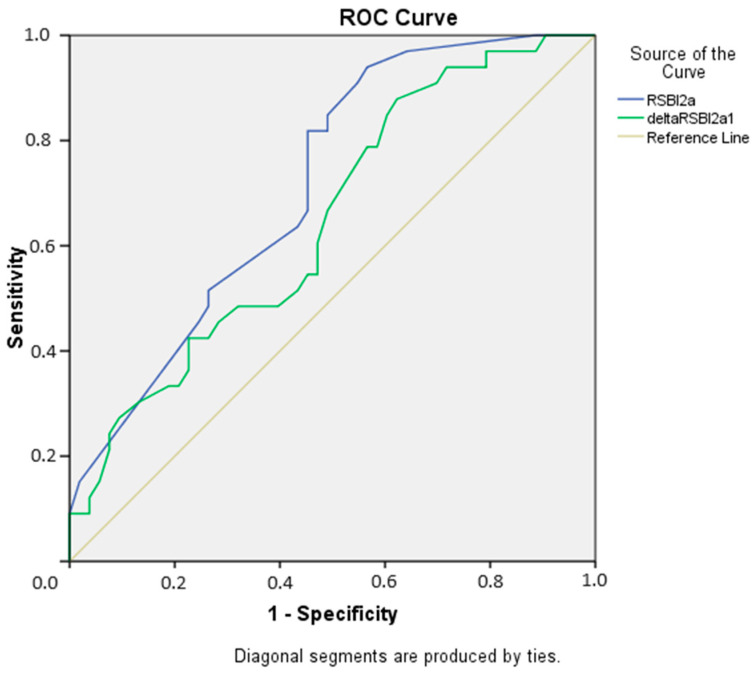
ROC curve of RSBI-2a (Rapid Shallow Breathing Index) and ΔRSBI variables in predicting extubation failure.

To find the optimal RSBI-2a and ΔRSBI cut-off values that distinguish the two groups, ROC analysis was performed, calculating the area under the curve (AUC) and Youden indices. The AUC for RSBI-2a was found to be 0.715 (0.055) (95% CI 0.608–0.822, *p* = 0.001). For ΔRSBI, the AUC was calculated as 0.648 (0.060) (95% CI 0.530–0.765, *p* = 0.022) (Figure 1). The optimal cut-off values, along with sensitivity, specificity, positive predictive value, negative predictive value, and test accuracy, are summarized in Table 4.

The optimal RSBI-2a cut-off value distinguishing the two groups was determined to be ≤72, with a sensitivity of 43.4%, specificity of 93.9%, positive predictive value of 92.0%, negative predictive value of 50.8%, and test accuracy of 62.79% (OR 11.883, 95% CI 2.574–54.858, *p* < 0.001). The optimal ΔRSBI cut-off value was determined to be ≤−3, with a sensitivity of 37.7%, specificity of 87.9%, positive predictive value of 83.3%, negative predictive value of 46.8%, and test accuracy of 56.98% (OR 4.394, 95% CI 1.345–14.354, *p* = 0.013).

Having an RSBI-2a value of 72 or lower before extubation increased the likelihood of successful extubation by approximately 10.8 times, while a ΔRSBI value of −3 or lower (indicating a reduction of 3 or more in RSBI-2a compared to RSBI-1) increased this likelihood by approximately 3.4 times. Using both cut-off values as criteria for positive extubation prediction showed a sensitivity of 30.2%, specificity of 100%, positive predictive value of 100%, negative predictive value of 47.1%, and test accuracy of 56.97% (OR 29.48, 95% CI 3.924–221.469, *p* < 0.001; * approximate value calculated using the Haldane–Anscombe method). Having both criteria positive simultaneously increased the likelihood of successful extubation by 28.48 times.

## 4. Discussion

In this study, the success rates of SBTs and extubation in patients receiving IMV support in the ICU at Hitit University were examined. A total of 86 patients were included, with 53 (61.6%) resulting in successful extubation and 33 (38.4%) resulting in unsuccessful extubation. There was no significant difference in RSBI-1 values between the groups; however, significant differences were observed in RSBI-1a, RSBI-2, and RSBI-2a values. The best cut-off value for RSBI-2a was found to be ≤72, which increased the likelihood of successful extubation by approximately 10.8 times. Additionally, a ΔRSBI ≤ −3 increased the likelihood of successful extubation by approximately 3.4 times.

Ventilator settings can alter RSBI measurements. The method of measurement, apart from the ventilator settings, can also affect the RSBI value obtained. A survey study found that some respiratory therapists use continuous positive airway pressure (CPAP) to measure RSBI, while others prefer pressure support ventilation (PSV) [12]. Shingala et al. compared RSBI measured during PSV with RSBI during spontaneous breathing and found that RSBI during PSV was more predictive of extubation, easier to perform, and more comfortable for patients [13]. Moreover, it is known that conditions such as sepsis, high body temperature, and patient positioning can increase respiratory rate and affect RSBI. Other factors, such as the size of the endotracheal tube and gender, can also influence RSBI, necessitating various threshold values [14]. In our study, RSBI measurements were performed using two different methods and compared.

The threshold value of RSBI is a controversial topic and has been examined in various studies. In one study, 304 patients were divided into groups where RSBI was measured but not used for weaning decisions, while in another group, RSBI was measured and a threshold of 105 breaths/min/L was used for weaning decisions [15]. The study found that weaning duration was significantly shorter when the weaning predictor was not used. Purro et al. found that the best cut-off value was 62 breaths/min/L and that the classical cut-off value (105 breaths/min/L) was inadequate [16]. Danaga et al. reported that the classical cut-off value of RSBI predicted only 20% of cases ready for extubation, but a cut-off value of 76.5 breaths/min/L provided a significant improvement in sensitivity [17]. Chao et al. determined the optimal RSBI threshold to be 97 breaths/min/L [18]. A retrospective cohort study of patients receiving prolonged IMV found that only the average RSBI and RSBI on the day of separation predicted success, while a single RSBI measurement did not predict successful separation from prolonged ventilation [19]. In our study, the predictive value of a single RSBI measurement before weaning was low, but significant differences were observed in RSBI-1a, RSBI-2, and RSBI-2a values. The optimal cut-off value for RSBI-2a was determined to be ≤72.

Although RSBI is suitable for most ICU patients, there are certain patient populations for whom RSBI may not accurately predict successful weaning. A study in patients with traumatic brain injury found that RSBI did not differ between successful and unsuccessful extubation groups [20]. Additionally, a study in patients with Chronic Obstructive Pulmonary Disease (COPD) found that RSBI measured during an early SBT did not accurately predict successful outcomes [21]. This is likely related to ineffective inspiratory efforts that do not trigger the ventilator. Oribabor et al. found that RSBI significantly shortened average extubation times in a group with cardiac disease [22]. In our study, it was found that the extubation success rate was lower in patients with pneumonia and COPD compared to other groups.

The RSBI ratio expresses the rate of change in RSBI in sequential measurements [23]. Segal et al. measured RSBI periodically during a 2 h SBT and expressed these values using a specific formula [(RSBI-2 − RSBI-1)/RSBI-1 × 100] [24]. They found that an RSBI ratio < 20% predicted weaning success with a sensitivity of 90.4% and a specificity of 100%. In another study by the same author, RSBI was evaluated every 30 min during a 2 h trial, and the initial RSBI was similar in both successful and unsuccessful extubation groups. RSBI values showed an increasing trend in the unsuccessful extubation group. This was attributed to an increasing respiratory rate or decreasing tidal volume. The study concluded that the percentage change in RSBI during SBT was a better predictor of extubation success than a single RSBI determination [25]. Another study also found the rate of change of RSBI between the 5th and 120th minutes was moderately more accurate than the single value of RSBI measured at the 120th minute in predicting extubation outcome [26]. In our study, intermittently measured RSBI values were compared among themselves, and the most important finding was that if there was a difference of more than −3 between the first and last measured value and the initial RSBI value was below 72, the extubation success rate was 100%. This indicates that serial measurements of RSBI are more predictive of successful extubation than a single measurement.

Our study has several important limitations. Firstly, the number of patients included in the study may be limited in terms of generalizing the results. Additionally, potential biases in patient selection may affect the accuracy of the results. For example, excluding patients with neuromuscular diseases, obesity, or heart failure limits the generalizability of the findings to these patient groups. Possible errors in the methods used for RSBI measurements may also affect the reliability of the results.

## 5. Conclusions

Our findings indicate that RSBI-2a and ΔRSBI values are significant predictors of extubation success. RSBI-2a ≤ 72 and ΔRSBI ≤ −3 significantly increase the likelihood of successful extubation. These results support the use of RSBI measurements in clinical practice. Similar studies have reported that RSBI is an important parameter in predicting extubation success. However, given the limitations of our study, our results should be interpreted with caution. Our findings need to be validated in other studies and different patient groups.

## Figures and Tables

**Table 1:** Participants’ baseline demographic and clinical data, alignment.

Demographic Data	Success (*n* = 53)	Failure (*n* = 33)	Total (*n* = 86)	*p*-Value
Gender (*n*%)				
Female	22	10	32 (37.2)	0.584
Male	31	23	54 (62.8)	
Age (years)				
Mean ± SD	56.15 ± 11.3	51.88 ± 12.81	54.51 ± 12.1	0.268

**Table 2:** Coexisting diseases of the patients, alignment.

Disease	Frequency (%)
Pneumonia	34.9
Heart failure	20.9
COPD exacerbation	14.0
Sepsis	10.5
Other	19.8

**Table 3:** The measurement results of the Rapid Shallow Breathing Index (RSBI) in patients.

	**Failure**					**Success**				
	**Median**	**Minimum**	**Maximum**	**Mean**	**SD**	**Median**	**Minimum**	**Maximum**	**Mean**	**SD**
RSBA-1	84.00	56.00	100.00	76.94	12.81	72.00	54.00	96.00	74.00	9.85
RSBI-1a	85.00	54.00	100.00	81.18	10.86	76.00	54.00	96.00	75.60	11.11
RSBI-2	85.00	70.00	102.00	85.45	6.48	80.00	54.00	102.00	79.38	10.81
RSBI-2a	92.00	65.00	120.00	91.00	10.48	80.00	54.00	97.00	78.83	14.72

RSBI: Rapid Shallow Breathing Index, SD: standard deviation.

**Table 4:** ROC Analysis for RSBI-2a and ΔRSBI, alignment.

Cut-Off Value	Sensitivity (%)	Specificity (%)	PPV (%)	N (%)	Test Accuracy (%)	OR (95% CI)	*p*-Value
RSBI-2a ≤ 72	43.4	93.9	92	50.8	62.79	11.883 (2.574–54.858)	<0.001
ΔRSBI ≤ −3	37.7	87.9	83.3	46.8	56.98	4.394 (1.345–14.354)	0.013
Both Criteria	30.2	100	100	47.1	56.97	29.48 * (3.924–221.469)	<0.001

* Approximate value calculated using the Haldane–Anscombe method. PPV: positive predictive value, NPV: negative predictive value.

## Data Availability

The data that support the findings of this study are not on a public domain because of privacy reasons but are available from the corresponding author upon reasonable request.
